# Temporal trends and barriers for inpatient palliative care referral in metastatic gynecologic cancer patients receiving specific critical care therapies

**DOI:** 10.3389/fonc.2023.1173438

**Published:** 2023-10-19

**Authors:** Li Shen, Longpei Chen, Yun Zhou, Tianran Chen, Hedong Han, Qiuyan Xia, Zhanguo Liu

**Affiliations:** ^1^ Department of Oncology, the Affiliated Aoyang Hospital of Jiangsu University, Zhangjiagang, China; ^2^ Department of Oncology, Changhai Hospital, Naval Medical University, Shanghai, China; ^3^ Department of Radiation Oncology, the Affiliated Aoyang Hospital of Jiangsu University, Zhangjiagang, China; ^4^ Department of Health Statistics, Naval Medical University, Shanghai, China

**Keywords:** trends, barriers, palliative care, metastatic gynecologic cancer, critical care therapies

## Abstract

**Objective:**

Existing evidence suggests that palliative care (PC) is highly underutilized in metastatic gynecologic cancer (mGCa). This study aims to explore temporal trends and predictors for inpatient PC referral in mGCa patients who received specific critical care therapies (CCT).

**Methods:**

The National Inpatient Sample from 2003 to 2015 was used to identify mGCa patients receiving CCT. Basic characteristics were compared between patients with and without PC. Annual percentage change (APC) was estimated to reflect the temporal trend in the entire cohort and subgroups. Multivariable logistic regression was employed to explore potential predictors of inpatient PC referral.

**Results:**

In total, 122,981 mGCa patients were identified, of whom 10,380 received CCT. Among these, 1,208 (11.64%) received inpatient PC. Overall, the rate of PC referral increased from 1.81% in 2003 to 26.30% in 2015 (APC: 29.08%). A higher increase in PC usage was found in white patients (APC: 30.81%), medium-sized hospitals (APC: 31.43%), the Midwest region (APC: 33.84%), and among patients with ovarian cancer (APC: 31.35%). Multivariable analysis suggested that medium bedsize, large bedsize, Midwest region, West region, uterine cancer and cervical cancer were related to increased PC use, while metastatic sites from lymph nodes and genital organs were related to lower PC referral.

**Conclusion:**

Further studies are warranted to better illustrate the barriers for PC and finally improve the delivery of optimal end-of-life care for mGCa patients who receive inpatient CCT, especially for those diagnosed with ovarian cancer or admitted to small scale and Northeast hospitals.

## 1 Introduction

Gynecologic cancer is the most common malignancy in women, encompassing ovarian cancer, uterine cancer, and cervical cancer. According to the Cancer Statistics for 2022, it is estimated that there will be approximately 19,880 new cases of ovarian cancer, 65,950 new cases of uterine cancer, and 14,100 new cases of cervical cancer in the United States (US). Meanwhile, the estimated deaths for gynecologic cancer are also less than encouraging (1). Early diagnosis and treatment could improve cancer survival, while a significant number of cases progress rapidly and are diagnosed with metastasis (1). For those admitted to intensive care units, patients are frequently administrated with critical care therapies (CCT) to provide respiratory and nutritional support for life-saving measures (2–4). These patients are usually experience severe physical, psychological and social suffering (5, 6).

Palliative care (PC) is a structured system that provides care to patients with end-stage diseases. It has been reported to improve symptom management, alleviate psychological suffering, and reduce cancer-related mortality (7). The American Society of Clinical Oncology (ASCO) and the Society of Gynecologic Oncology (SGO) have formally endorsed early palliative care for gynecologic cancer patients (8–10). Multiple studies have demonstrated the beneficial role of early PC in addressing symptoms and managing psychological concerns in patients with gynecologic oncology (11, 12). However, studies have reported that PC is highly underutilized in metastatic gynecologic cancer (mGCa) patients, with utilization rates ranging from 5% to 24% (13–17). mGCa patients receiving CCT have increased cancer-related complications and long-term morbidity, and thus are strong indications for PC referral (18). Increasing awareness and accessibility of PC in this population is clinically significant. Although several publications have examined the utilization pattern of inpatient PC across different cancers in patients receiving life-sustaining treatments (19–21), there is a dearth of data focusing specifically on PC referral in mGCa patients receiving CCT while hospitalized.

The present study aims to investigate the temporal trends, predictors and barriers for inpatient PC referral in mGCa patients who specific CCT from a national perspective using the National Inpatient Sample (NIS) database.

## 2 Patients and methods

### 2.1 Data source

Data in the study is de-identified and thus exempt from approval by an institutional review board. The NIS database is the largest publically available all-payer healthcare database in the US (22), developed by the Agency for Healthcare Research and Quality (AHRQ), as part of the Healthcare Cost and Utilization project (HCUP), which collected a stratified sample from nearly 1000 hospitals. Each hospitalization contains up to 30 inpatient diagnoses and 15 procedures that could be identified through the International Classification of Diseases, Ninth Revision, Clinical Modification (ICD-9-CM) codes.

### 2.2 Study design and patient selection

NIS database from 2003 to 2015 was used in this cross-sectional study. Gynecologic cancers were obtained by retrieving the following diagnostic codes: 1830, 1832, 1838, 1839 (ovarian cancer), 179, 1820, 1821, 1828 (uterine cancer), 1800, 1801, 1808, 1809 (cervical cancer) (16). Cases were considered metastatic gynecologic cancer (mGCa) with the presence of bone & bone marrow, brain & spinal cord, lymph nodes, liver, respiratory organs, urinary organs, adrenal glands, gastrointestinal organs, genital organs or other organs in the field of the secondary codes (**Supplementary Table 1**) (23). Among the selected mGCa cases, specific CCT including invasive mechanic ventilation (IMV), total parenteral nutrition (TPN), percutaneous endoscopic gastrostomy (PEG) tube, tracheostomy and dialysis for acute kidney failure (AKF) were considered (19–21). These procedures are aggressive and commonly used during the end-of-life period to provide necessary respiratory and nutritional support.

### 2.3 Patient and hospital characteristics

Patient-related, cancer-related and hospital-related characteristics were collected. Patient-related characteristics included age, year of admission, race, insurance type, income category, discharge destination, primary diagnosis and Elixhauser comorbidity score. The last consisted of 29 common comorbidities that could represent the disease burdens (excluded cancer in this study) (24). Cancer-related characteristics encompassed cancer type, metastatic sites, number of metastatic sites and chemotherapy. Lastly, hospital-related characteristics were hospital type, hospital bedsize and hospital region.

### 2.4 Definition of principal diagnosis and inpatient PC use

The principal diagnosis was categorized using the Clinical Classifications Software codes, which collapsed diagnoses and procedures into clinically meaningful categories (22).The primary outcome was temporal trend of inpatient PC referral in mGCa patients who received specific CCT. The secondary outcome included predictors of PC referral in the overall patients and in the subgroup undergoing IMV treatment. PC referral was defined using ICD-9-CM diagnostic code V66.7, which has been validated in metastatic disease with moderate sensitivity and high specificity (25, 26). Cases involving patients under 18 years old or admitted to hospitals that did not provide PC service during the study period were excluded from the analysis.

### 2.5 Statistical analysis and covariates

Continuous characteristics between patients with and without PC referral were expressed as mean and compared using t-test, while categorical variables were reported as proportions and compared using chi-square tests. We calculated annual percentage change (APC) in the entire cohort and subgroups by race, hospital region, hospital bedsize, teaching status, cancer type and discharge destination. Sampling stratas, clusters and weights were considered to derive estimates from the national perspective using complex survey methods. Additionally, we preformed multivariable logistic regression analysis to explore the predictors of PC referral in mGCa patients receiving CCT, taking into account patient-related, cancer-related and hospital-related characteristics. Confidence intervals for the ORs were calculated using the Taylor series method.

A P value ≤ 0.05 was considered statistically significant. All statistical analyses were performed using SAS version 9.4 and R version 3.6.2.

## 3 Results

### 3.1 Study population

In total, 122,981 hospitalizations diagnosed with mGCa were identified from 2003 to 2015, among which 10,737 have received inpatient CCT. We further excluded 357 patents who were under 18 years old or admitted to hospitals where PC was not available. Consequently, 10,380 (weighted 51,008) mGCa patients receiving CCT were identified in the further analysis. Among these patients, 7,254 (69.88%) were diagnosed with metastatic ovarian cancer (mOCa), 1,931 (18.60%) were diagnosed with metastatic uterine cancer (mUCa) and 1,195 (11.51%) were diagnosed with metastatic cervical cancer (mCCa). Regarding specific CCT, 3,641 (35.08%) patients received IMV, 1,207 (11.63%) received PEG, 5,918 (57.01%) received TPN, 265 (2.55%) received tracheostomy and 695 (6.70%) received dialysis for AKF. Characteristics between patients with and without PC in the IMV subgroup were summarized in **Supplementary Table 2**.

### 3.2 Trends of IPC use

**Figure 1:**
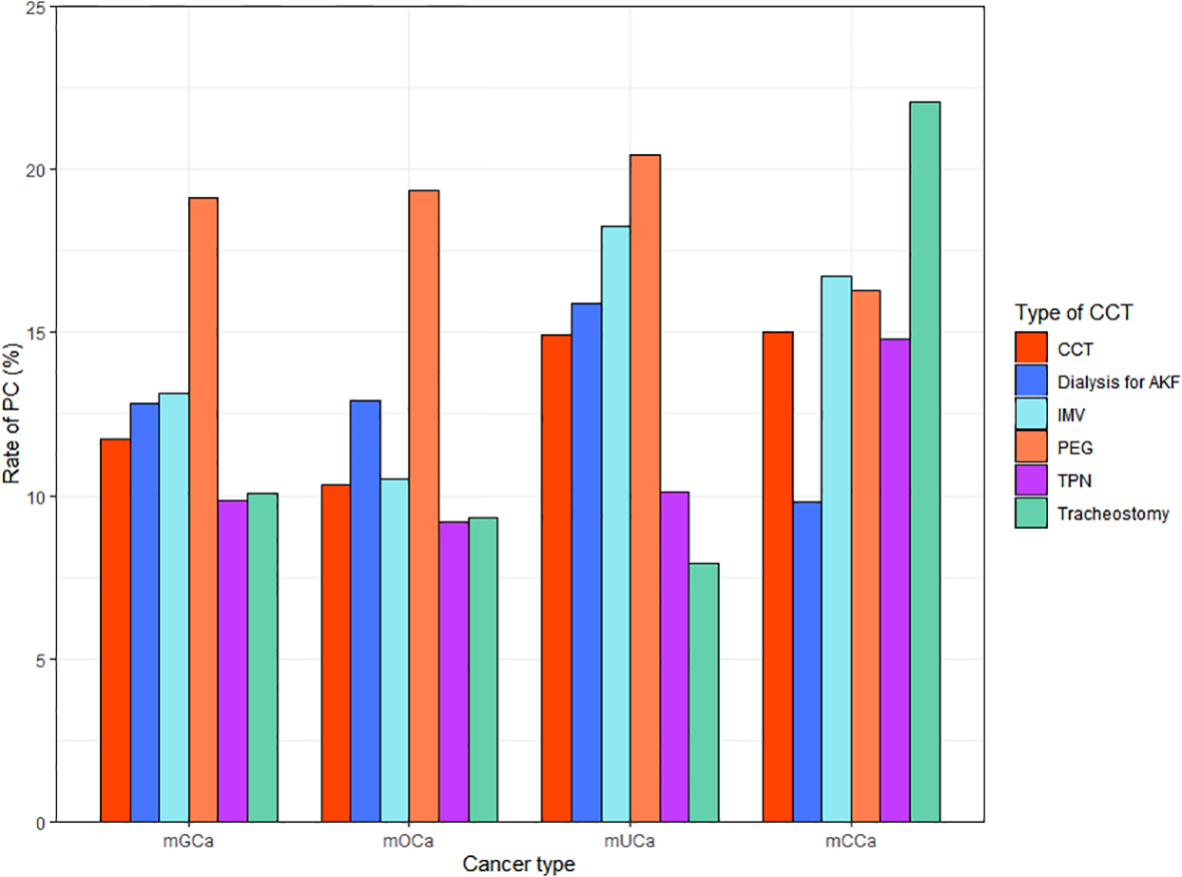
Rate of palliative care referral by CCT and cancer type.

Among the included patients, 1208 (11.64%) received inpatient PC. There were 743(10.24%), 288 (14.91%) and 177(14.81%) patients who received PC in mOCa, mUCa and mCCa patients, respectively. As showed in **Figure 1**, the rates of PC referral varied across different types of CCT and cancer. Patients who received PC were younger (61.74 vs. 62.92), less likely to be diagnosed with mOCa (61.51% vs. 70.99%), more likely to be admitted for infections (14.32% vs. 8.18%) and admitted in Midwest (22.60% vs. 19.41%) or urban teaching hospitals (69.62% vs. 62.91%) (**Table 1**).

**Table 1:** Basic characteristics of mGCa patients receiving CCT stratified according to use of inpatient PC.

Variables	No PC (N=9172, %)	PC (N=1208, %)	*P*-value
Age	62.92 (12.65)	061.74 (13.14)	0.0024
Year interval			<0.0001
2003-2009	5046 (55.02)	228 (18.87)	
2010-2014	4126 (44.98)	980 (81.13)	
Race			<0.0001
White	5775 (62.97)	741 (61.34)	
Black	1013 (11.04)	205 (16.97)	
Hispanic	651 (7.10)	102 (8.44)	
Other	544 (5.93)	81 (6.71)	
Unknown	1189 (12.96)	79 (6.54)	
Type of insurance			
Medicare	4288 (46.75)	534 (44.21)	0.0004
Medicaid	1018 (11.10)	177 (14.65)	
Private	3452 (37.64)	427 (35.35)	
Self-pay/other	414 (4.51)	70 (5.79)	
Income quartile			0.2463
0-25^th^ Percentile	1985 (21.64)	282 (23.34)	
25^th^-50^th^ Percentile	2043 (22.27)	277 (22.93)	
50^th^-75^th^ Percentile	2406 (26.23)	287 (23.76)	
75^th^-100^th^ Percentile	2738 (29.86)	362 (29.97)	
Hospital bedsize			0.0008
Small	951 (10.36)	86 (7.12)	
Medium	1945 (21.21)	285 (23.59)	
Large	6276 (68.43)	837 (69.29)	
Hospital type			<0.0001
Rural	358 (3.90)	55 (4.55)	
Urban non-teaching	3044 (33.19)	312 (25.83)	
Urban teaching	5770 (62.91)	841 (69.62)	
Hospital region			0.0124
Northeast	2063 (22.49)	232 (19.21)	
Midwest	1780 (19.41)	273 (22.60)	
South	3012 (32.84)	403 (33.36)	
West	2317 (25.26)	300 (24.83)	
Elixhauser comorbidity score	2.71 (1.78)	3.11 (1.73)	<0.0001
Primary diagnosis			<0.0001
Cancer-related disorders	5080 (55.39)	550 (45.53)	
Infections	750 (8.18)	173 (14.32)	
Genitourinary disorders	394 (4.30)	47 (3.89)	
Cardiovascular disorders	294 (3.21)	51 (4.22)	
Pulmonary disorders	436 (4.75)	74 (6.13)	
Gastrointestinal disorders	1390 (15.15)	188 (15.56)	
Fractures	*	*	
Fluid/Electrolyte disorders	121 (1.32)	23 (1.90)	
Neurologic disorders	75 (0.82)	14 (1.16)	
Complications of surgery	346 (3.77)	53 (4.39)	
Other disorders	278 (3.03)	31 (2.57)	
Cancer type			<0.0001
Ovarian cancer	6511 (70.99)	743 (61.51)	
Uterine cancer	1643 (17.91)	288 (23.84)	
Cervical cancer	1018 (11.10)	177 (14.65)	
Metastatic sites			
Bone & bone marrow	423 (4.61)	99 (8.20)	<0.0001
Brain & spinal cord	256 (2.79)	65 (5.38)	<0.0001
Lymph nodes	1499 (16.34)	155 (12.83)	0.0017
Liver	1747 (19.05)	336 (27.81)	<0.0001
Respiratory organs	2211 (24.11)	342 (28.31)	0.0014
Adrenal glands	64 (0.70)	13 (1.08)	0.1497
Gastrointestinal organs	5637 (61.46)	659 (54.55)	<0.0001
Urinary organs	529 (5.77)	66 (5.46)	0.6692
Genital organs	1088 (11.86)	61 (5.05)	<0.0001
Other organs	1881 (20.51)	195 (16.14)	0.0004
Number of metastatic sites (≥2)	3988 (43.48)	515 (42.63)	0.5763
Type of CCT			
IMV	3166 (34.52)	475 (39.32)	0.0010
PEG tube	977 (10.65)	230 (19.04)	<0.0001
TPN	5342 (58.24)	576 (47.68)	<0.0001
Tracheostomy	239 (2.61)	26 (2.15)	0.3476
AKI requiring dialysis	606 (6.61)	89 (7.37)	0.3202
Do Not Resuscitate	442 (4.82)	442 (36.59)	<0.0001
Chemotherapy	1041 (11.35)	122 (10.10)	0.1953
In-hospital mortality	1821 (19.85)	523 (43.29)	<0.0001
Discharge disposition (alive)			<0.0001
Home or home healthcare	5131 (69.80)	410 (59.85)	
Short term hospitals	268 (3.65)	17 (2.48)	
Intermediate facilities	1925 (26.19)	250 (36.50)	
Other	27 (0.36)	*	

CCT, critical care therapies; mGCa, metastatic gynecologic cancer; PC, palliative care; SD, standard deviation; IMV, invasive mechanic ventilation; PEG, percutaneous endoscopic gastrostomy; TPN, total parenteral nutrition; AKF, acute kidney failure.

*Small numbers of observations (<10) are at risk of identification of persons according to the HUCP and we replaced the number with an asterisk.

**Figure 2:**
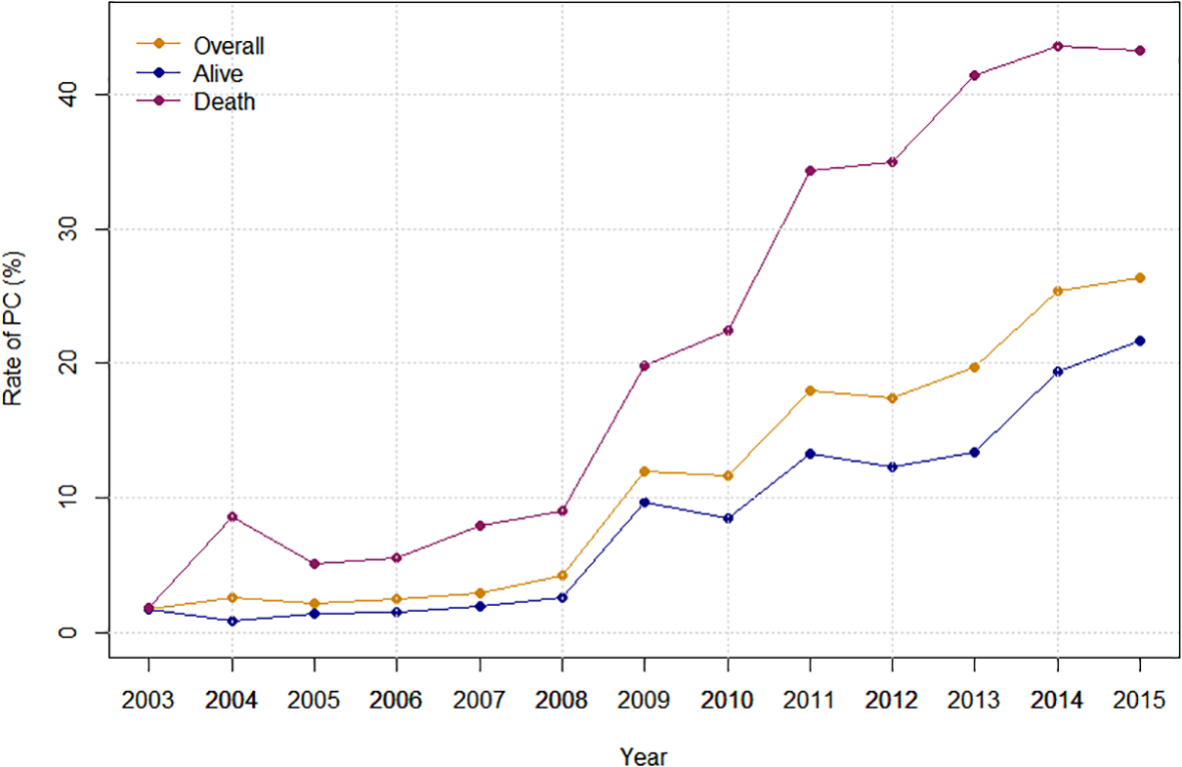
Inpatient palliative care referral over time, stratified by hospital region, race, hospital bedsize and hospital teaching status.

**Figure 3:**
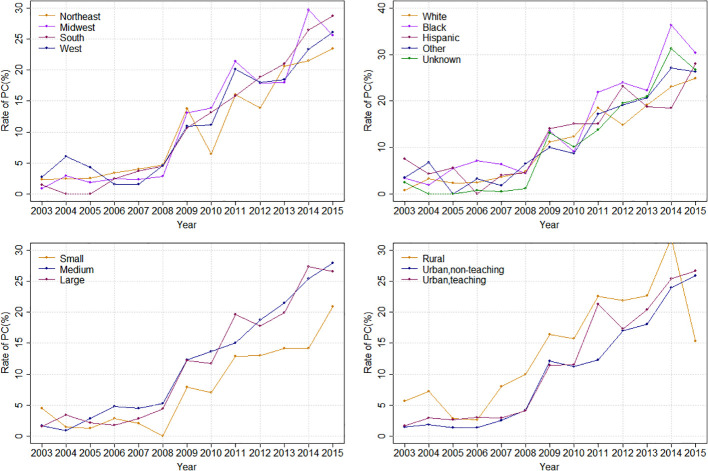
Inpatient palliative care referral over time, stratified by cancer type.

Overall, the rate of PC referral increased from 1.81% in 2003 to 26.30% in 2015 (APC: 29.08%%; p < 0.0001). Stratified by race, the PC rate increased from 0.78% to 24.93% in White (APC: 30.81%%; p < 0.0001), from 3.33% to 30.43% in Black (APC: 24.92%%; p < 0.0001) and from 7.47% to 28.07% (APC: 16.48%%; p=0.0005) in the Hispanic population (**Figure 2**). Stratified by bedsize, the PC rate increased from 4.46% to 20.90% in small bedsize hospitals (APC: 23.99%; p=0.0001), from 1.60% to 27.97% in medium bedsize hospitals (APC: 31.43%%; p < 0.0001) and from 1.53% to 26.63% in large bedsize hospitals (APC: 30.55%%; p < 0.0001). Stratified by hospital region, the PC rate increased from 2.29% to 23.48% in the Northeast (APC: 24.92%; p < 0.0001), from 0.87% to 25.62% in the Midwest (APC: 33.84%; p < 0.0001), from 1.40% to 28.72% in the South (APC: 31.88%; p < 0.0001) and from 2.71% to 26.15% in the West (APC: 24.35%; p=0.0004). In addition, stratified by cancer type, the rate of PC referral increased from 1.06% to 23.32% in mOCa (APC: 31.35%; p < 0.0001), from 3.06% to 33.58% in mUCa (APC: 27.68%; p < 0.0001), and from 4.81% to 28.17% in mCCa (APC: 25.80%; p < 0.0001; **Figure 3**). Stratified by discharge destination, the PC rate increased from 1.79% to 21.63% in patients who died during hospitalization (APC: 32.00%; p < 0.0001) and from 1.88% to 43.20% among the survivors (APC: 27.78%; p < 0.0001; **Supplementary Figure 1**).

### 3.3 Predictors of PC use

According to the multivariable analysis, year interval (odds ratio[OR]: 2.87, 95% confidence interval [CI]: 2.33-3.55), medium bedsize (OR: 1.59, 95% CI: 1.17-2.17), large bedsize (OR: 1.59, 95% CI: 1.20-2.12), Midwest region (OR: 1.37, 95% CI: 1.06-1.78), West region (OR: 1.30, 95% CI: 1.01-1.66), higher Elixhauser comorbidity score (OR: 1.04, 95% CI: 1.00-1.08), uterine cancer (OR: 1.28, 95% CI: 1.09-1.51), cervical cancer (OR: 1.38, 95% CI: 1.10-1.74), Do Not Resuscitate (OR: 6.20, 95% CI: 5.22-7.37), patients receiving PEG tube (OR: 1.98, 95% CI: 1.57-2.51), metastatic sites from brain & spinal cord (OR: 1.58, 95% CI: 1.10-2.28) and liver (OR: 1.34, 95% CI: 1.07-1.67) were associated with increased PC referral, while urban non-teaching hospitals, metastatic sites from lymph nodes and genital organs were related to lower PC referral. Additionally, predictors of PC referral in patients receiving IMV could be found in **Table 2**, which were similar to results in the main analysis.

**Table 2:** Predictors of PC use in mGCa patients receiving CCT and IMV.

Variables	CCT		IMV	
	OR (95%CI)	*P*-value	OR (95%CI)	*P*-value
Age	1.00 (0.99,1.00)	0.2391	1.00 (0.98,1.01)	0.4806
Year interval				
2003-2009	1.00		1.00	
2010-2015	2.87 (2.33,3.55)	<0.0001	3.25 (2.33,4.54)	<0.0001
Race				
White	1.00		1.00	
Black	1.23 (0.99,1.51)	0.0587	1.20 (0.88,1.62)	0.2479
Hispanic	0.95 (0.72,1.25)	0.7184	0.84 (0.55,1.29)	0.4363
Other	1.07 (0.83,1.39)	0.6080	0.96 (0.63,1.47)	0.8508
Unknown	0.68 (0.50,0.93)	0.0156	0.68 (0.43,1.08)	0.0989
Type of insurance				
Medicare	1.00		1.00	
Medicaid	1.05 (0.82,1.35)	0.7069	0.96 (0.66,1.41)	0.8417
Private	0.98 (0.81,1.19)	0.8607	1.02 (0.75,1.39)	0.8773
Self-pay/other	1.21 (0.86,1.72)	0.2778	0.79 (0.44,1.42)	0.4280
Income quartile				
0-25^th^ Percentile	1.00		1.00	
25^th^-50^th^ Percentile	1.12 (0.91,1.37)	0.2928	1.21 (0.88,1.68)	0.2442
50^th^-75^th^ Percentile	1.00 (0.81,1.22)	0.9771	1.09 (0.79,1.50)	0.6176
75^th^-100^th^ Percentile	1.23 (1.00,1.52)	0.0529	1.36 (0.98,1.89)	0.0646
Hospital bedsize				
Small	1.00		1.00	
Medium	1.59 (1.17,2.17)	0.0033	1.74 (1.05,2.88)	0.0331
Large	1.59 (1.20,2.12)	0.0014	1.83 (1.15,2.92)	0.0108
Hospital type				
Rural	1.00		1.00	
Urban non-teaching	0.65 (0.45,0.93)	0.0201	0.56 (0.33,0.98)	0.0415
Urban teaching	0.87 (0.61,1.23)	0.4332	0.86 (0.51,1.45)	0.5604
Hospital region				
Northeast	1.00		1.00	
Midwest	1.37 (1.06,1.78)	0.0181	1.23 (0.83,1.83)	0.3015
South	1.13 (0.89,1.43)	0.3072	1.04 (0.73,1.46)	0.8390
West	1.30 (1.01,1.66)	0.0399	1.24 (0.85,1.80)	0.2664
Elixhauser comorbidity score	1.04 (1.00,1.08)	0.0486	1.00 (0.93,1.06)	0.9017
Primary diagnosis				
Cancer-related disorders	1.00		1.00	
Infections	1.11 (0.88,1.41)	0.3882	1.37 (0.99,1.89)	0.0546
Genitourinary disorders	0.86 (0.60,1.25)	0.4358	1.34 (0.59,3.04)	0.4853
Cardiovascular disorders	1.01 (0.72,1.43)	0.9373	1.58 (1.03,2.41)	0.0355
Pulmonary disorders	1.11 (0.82,1.51)	0.4833	1.41 (0.96,2.07)	0.0778
Gastrointestinal disorders	0.93 (0.75,1.14)	0.4728	1.65 (1.03,2.63)	0.0369
Fractures	3.47 (0.69,17.37)	0.1306	5.01 (1.02,24.59)	0.0469
Fluid/Electrolyte disorders	1.71 (1.06,2.77)	0.0293	5.01 (1.31,19.19)	0.0189
Neurologic disorders	1.15 (0.60,2.21)	0.6751	0.74 (0.20,2.82)	0.6639
Complications of surgery	1.35 (0.99,1.84)	0.0551	1.97 (1.09,3.57)	0.0255
Other disorders	0.78 (0.50,1.20)	0.2572	1.22 (0.63,2.34)	0.5541
Cancer type				
Ovarian cancer	1.00		1.00	
Uterine cancer	1.28 (1.09,1.51)	0.0033	1.80 (1.37,2.35)	<0.0001
Cervical cancer	1.38 (1.10,1.74)	0.0051	1.61 (1.12,2.32)	0.0102
Metastatic sites				
Bone & bone marrow	1.14 (0.85,1.54)	0.3759	0.96 (0.63,1.46)	0.8561
Brain & spinal cord	1.58 (1.10,2.28)	0.0130	0.96 (0.57,1.62)	0.8731
Lymph nodes	0.78 (0.63,0.98)	0.0290	0.70 (0.50,0.98)	0.0402
Liver	1.34 (1.07,1.67)	0.0103	0.93 (0.66,1.30)	0.6549
Respiratory organs	1.00 (0.83,1.21)	0.9845	0.86 (0.62,1.17)	0.3366
Adrenal glands	0.78 (0.39,1.58)	0.4936	0.63 (0.25,1.63)	0.3427
Gastrointestinal organs	0.98 (0.80,1.19)	0.8305	1.05 (0.77,1.42)	0.7660
Urinary organs	1.03 (0.75,1.40)	0.8718	0.74 (0.43,1.26)	0.2662
Genital organs	0.58 (0.42,0.78)	0.0004	0.55 (0.34,0.91)	0.0187
Other organs	0.88 (0.71,1.09)	0.2369	0.89 (0.63,1.27)	0.5298
Number of metastatic sites (≥2)	0.98 (0.77,1.25)	0.8951	1.29 (0.88,1.89)	0.1855
Type of CCT				
IMV	1.14 (0.90,1.43)	0.2807	—	—
PEG tube	1.98 (1.57,2.51)	<0.0001	1.38 (0.63,2.98)	0.4201
TPN	1.05 (0.84,1.32)	0.6639	1.04 (0.74,1.47)	0.8216
Tracheostomy	0.76 (0.46,1.25)	0.2801	0.84 (0.49,1.43)	0.5193
AKI requiring dialysis	0.99 (0.72,1.36)	0.9513	1.01 (0.61,1.66)	0.9700
Chemotherapy	1.05 (0.84,1.31)	0.6697	1.41 (0.89,2.22)	0.1445
Do Not Resuscitate	6.20 (5.22,7.37)	<0.0001	6.90 (5.27,9.03)	<0.0001

CCT, critical care therapies; mGCa, metastatic gynecologic cancer; PC, palliative care; IMV, invasive mechanic ventilation; PEG, percutaneous endoscopic gastrostomy; TPN, total parenteral nutrition; AKF, acute kidney failure; OR, odds ratio; CI, confidence interval.

## 4 Discussion

Although ASCO and SGO have long recommended early integration of PC to improve end-of-life care, practical evidence shows high underutilization of PC referral in mGCa patients (8, 10, 13, 16). Intensive care therapies are often provided to mGCa patients when severe treatment-related complications occurred or cancer progressed, highlighting the clinical importance and necessity of PC referral in this vulnerable population (2, 18). Our analysis suggested that approximately 11.64% of patients received inpatient PC, and the rate of PC referral increased from 1.81% in 2003 to 26.30% in 2015, with an average annual increase of 29.08%. Multivariable analysis suggested that medium bedsize, large bedsize, Midwest region, West region, higher Elixhauser comorbidity score, uterine cancer and cervical cancer were related to increased PC use, while urban non-teaching hospitals, metastatic sites from lymph nodes and genital organs were related to lower PC referral.

Overall, approximately 11.64% of mGCa patients with CCT received inpatient PC, which is more than two times higher than the reported PC rate of 5% in the entire population regardless of CCT, as reported by Rosenfeld et al. (13). However, this proportion is still far from satisfactory considering that all mGCa patients with CCT are candidates for PC referral. It is worth noting that PC referral consistently increased by 29.08% from 2003 to 2015. This phenomenon might reflect improved adherence of oncological guideline by both physicians and patients. Subgroup analysis indicated that increasing trend of PC referral was more pronounced in White and patients admitted to medium bedsize, urban non-teaching and Midwest hospitals, suggesting a wider acceptance of PC use in these patients. From the trend charts, it is evident that PC rate experienced a sharp increase since 2009, which aligns with the findings of previous publications (13, 16). As aggressive measures such as CCT can reduce quality of life in mGCa patients, this unexpected increase may be partly attributed to the landmark ENABLE II trial in 2009 that revealed the effectiveness of PC interventions in improving the quality of life for patients with advanced cancer (27).

When considering hospital region, patients hospitalized in Midwest hospitals had the highest PC rate (13.30%), followed by South (11.80%), West (11.46%) and Northeast (10.11%), accompanied by the highest APC (33.84%). Multivariable analysis accounting for potential confounders suggested that Midwest region (OR: 1.37) and West region (OR: 1.30) were associated with increased probability of PC referral compared to the Northeast region. This regional disparities in PC use has been previously reported. Milki et al. enrolled mGCa patients who subsequently died during hospitalization and found that patients in Midwest region (OR: 1.37) and West region (OR: 1.30) had increased PC use (16). Another study focusing on metastatic bladder cancer receiving CCT also described a higher PC rate in the West region (21). Further studies are warranted to understand the undelaying mechanisms for this geographic disparities and to relieve barriers for lower PC utilization in the Northeast region.

When considering hospital size, we observed that both medium bedsize (OR: 1.59) and large bedsize (OR: 1.59) were associated with increased PC use compared to small bedsize. One possible explanation for this finding might be that larger hospitals have more dedicated end-of-life specialists to provide PC services. However, research on this topic has produced conflicting results. For instance, Rosenfeld et al. conducted a study using data from the 2005 to 2011 NIS database, including all mGCa cases, and concluded that bedsize was not a predictor for PC referral (13). Another study by Milki et al. found that large bedsize was a positive predictor of PC referral (OR: 1.36) in mGCa cases who died in hospital (16). We hypothesized that the severity of dying status might result this disparity, as mGCa patients receiving CCT or died in hospital represented more severe conditions with significant symptom burden. Large bedsize hospitals are likely to form well-organized PC team and well-established relationship between physicians and mGCa patients with more severe conditions.

There has been controversy surrounding the emerging evidence on racial disparities in PC use among mGCa patients (2, 13–16). Understanding the racial and cultural differences among various racial groups can help personalize palliative care for mGCa patients receiving CCT and improve the delivery of comprehensive cancer care. Studies have reported Studies have reported that racial minority groups, such as Black or Hispanic gynecologic cancer patients, have expressed a desire for more intensive and invasive end-of-life care (2, 28), making them the potential candidates for PC delivery from the perspective of end-of-life decision-making. Consistent with previous publications (13, 29), our findings showed that Hispanic patients had the highest rate of PC use (16.83%), followed by Black patients (13.55%) and White patients (11.37%). However, this significant finding disappeared after adjustment for patient-related, cancer-related and hospital-related characteristics. Notably, Islam et al. analyzed data from the 2016 National Cancer Database and found that Hispanic and Black patients were less likely to utilize PC in metastatic ovarian cancer patients (14). In our subgroup analysis focusing exclusively on metastatic ovarian cancer patients, we did not observe such racial disparities. These discrepancies may be attributed to different population groups and data sources, especially considering that our study specifically involved patients receiving CCT during hospitalization. Therefore, further studies are needed to provide sufficient evidence to better understand the underlying racial differences and to improve equitable provision of PC among mGCa patients, irrespective of race.

For cancer types, uterine cancer ranked first in the rate of PC use (14.91%), followed by cervical cancer (14.81%) and ovarian cancer (10.24%). Although ovarian cancer patients has the lowest rate of PC use, the use of PC has dramatically increased over the study period, with the highest APC (31.35%). Previous studies have also reported lower PC use in ovarian cancer (13, 14). As we know, ovarian cancer has a higher degree of malignancy and worse survival compared to uterine cancer and cervical cancer (30). Therefore, future efforts are needed to improve and optimize PC referral in metastatic ovarian patients receiving CCT.

The present study utilized a national-level hospitalized database covering long time spans to investigate the temporal trends and predictors for inpatient PC referral in mGCa patients who frequently received CCT, including IMV, TPN, PEG tube, tracheostomy and dialysis for AKF. However, several limitations should also be considered for an accurate interpretation of our results. Firstly, PC use in the NIS database was defined based on the ICD-9-CM diagnostic code V66.7. Being an administrative database, the NIS may not capture all instances of PC discussions, and only those that are documented by physicians are recorded. Therefore, there may be a bias towards underestimating the actual number of PC use cases. However, the code was initially introduced in 1996 and has since been used in several publications, demonstrating moderate sensitivity (66.3% to 83%) and high specificity (95% to 99.1%) (25, 26). Secondly, this study focused only on specific CCTs that were frequently used in routine clinical practice. Any external extrapolation (eg, to all critically ill mGCa patients) should be interpreted with adequate caution. Thirdly, race information was unknown for nearly 12.22% of the included patients. Despite these limitations, the present study provides new evidence and insights into the understanding of PC referral in mGCa patients receiving CCT.

This analysis suggests that approximately 11.64% of patients received inpatient PC, which is still considerably below an ideal level. Further studies are necessary to elucidate the barriers to PC and ultimately enhance the provision of optimal end-of-life care for mGCa patients who receive inpatient CCT. This is particularly important for patients with ovarian cancer or those admitted to small-scale and northeast hospitals.

## 5 Conclusions

Despite the increase in PC referral over time, the absolute rate has remained low. The rates of PC referral in mGCa patients receiving CCT differ based on various sociodemographic and clinical factors. Thus, further studies are necessary to better understand the barriers to PC in mGCa patients undergoing inpatient CCT.

## Data availability statement

The datasets presented in this article are not readily available because data are available in the NIS website: www.hcup-us.ahrq.go. Requests to access the datasets should be directed to www.hcup-us.ahrq.gov.

## Ethics statement

The studies involving humans were approved by The NIS database is publically available and de-identified and thus is exempt from approval by an institutional review board in Aoyang Hospital of Jiangsu University. The studies were conducted in accordance with the local legislation and institutional requirements. The ethics committee/institutional review board waived the requirement of written informed consent for participation from the participants or the participants’ legal guardians/next of kin because The NIS database is publically available and de-identified and thus is exempt from approval by an institutional review board in Aoyang Hospital of Jiangsu University.

## Author contributions

LS, LC, YZ, HH, ZL designed the study and drafted the manuscript. TC, QX edited the manuscript. In addition, each author has read and approved the final version of the manuscript. All authors contributed to the article and approved the submitted version.
