# Supplementary material for: Temporal trends and barriers for inpatient palliative care referral in metastatic gynecologic cancer patients receiving specific critical care therapies
Source: Front Oncol. 2023 Oct 19;13:1173438. doi: 10.3389/fonc.2023.1173438 (PMC10620795; doi:10.3389/fonc.2023.1173438)
Supplement: Supplementary file 1 [file DataSheet_1.docx]

**Supplementary tables**

**Supplementary table 1. Diagnostic codes used in this study**

| **Disease Entity** | **Codes** |
| --- | --- |
| **Cancer type (ICD-9-CM codes)** |  |
| Ovarian cancer | 1830, 1832, 1838, 1839 |
| Uterine cancer | 179, 1820, 1821, 1828 |
| Cervical cancer | 1800, 1801, 1808, 1809 |
| **Primary diagnosis (CCS codes)** |  |
| Cancer-related disorders | 11-44 |
| Infections | 1-9 |
| Genitourinary disorders | 156-196 |
| Cardiovascular disorders | 96-121 |
| Pulmonary disorders | 122-134 |
| Gastrointestinal disorders | 135-155 |
| Fractures | 207, 228-231 |
| Fluid/Electrolyte disorders | 55 |
| Neurologic disorders | 76-85 |
| Complications of device or surgery | 237-238 |
| **Secondary tumor site (ICD-9-CM codes)** |  |
| Bone & bone marrow | 198.5 |
| Brain & spinal cord | 198.3, 198.4 |
| Lymph nodes | 196.x |
| Liver | 197.7 |
| Respiratory organs | 197.0, 197.1, 197.2, 197.3, 197.8 |
| Urinary organs | 198.0, 198.1 |
| Adrenal glands | 198.7 |
| Gastrointestinal organs | 197.4, 197.5, 197.6 |
| Genital organs | 198.82 |
| Other organs | 198.81, 198.89, 198.2 |

Abbreviation: CCS, Clinical Classifications Software; ICD-9-CM, International Classification of Diseases, Ninth Revision, Clinical Modification;

**Supplementary Table 2. Basic characteristics between mGCa patients receiving IMV with and without PC**

| **Variables** | **No PC (N=3166, %)** | **PC (N=475, %)** | ***P*-value** |
| --- | --- | --- | --- |
| **Age** | 63.71(12.68) | 62.17(13.34) | 0.0140 |
| **Year interval** |  |  | <0.0001 |
| 2003-2009 | 1687(53.28) | 73(15.37) |  |
| 2010-2014 | 1479(46.72) | 402(84.63) |  |
| **Race** |  |  | <0.0001 |
| White | 1841(58.15) | 266(56.00) |  |
| Black | 461(14.56) | 105(22.11) |  |
| Hispanic | 281(8.88) | 45(9.47) |  |
| Other | 241(7.61) | 32(6.74) |  |
| Unknown | 342(10.80) | 27(5.68) |  |
| **Type of insurance** |  |  | 0.1265 |
| Medicare | 1593(50.32) | 217(45.69) |  |
| Medicaid | 450(14.21) | 84(17.68) |  |
| Private | 948(29.94) | 150(31.58) |  |
| Self-pay/other | 175(5.53) | 24(5.05) |  |
| **Income quartile** |  |  | 0.9222 |
| 0-25^th^ Percentile | 789(24.92) | 122(25.68) |  |
| 25^th^-50^th^ Percentile | 744(23.50) | 115(24.21) |  |
| 50^th^-75^th^ Percentile | 775(24.48) | 110(23.16) |  |
| 75^th^-100^th^ Percentile | 858(27.10) | 128(26.95) |  |
| **Hospital bedsize** |  |  | 0.2433 |
| Small | 276(8.71) | 31(6.52) |  |
| Medium | 678(21.42) | 109(22.95) |  |
| Large | 2212(69.87) | 335(70.53) |  |
| **Hospital type** |  |  | 0.0039 |
| Rural | 118(3.73) | 22(4.63) |  |
| Urban non-teaching | 1060(33.48) | 123(25.89) |  |
| Urban teaching | 1988(62.79) | 330(69.47) |  |
| **Hospital region** |  |  | 0.2727 |
| Northeast | 724(22.87) | 97(20.42) |  |
| Midwest | 572(18.07) | 102(21.47) |  |
| South | 1061(33.51) | 160(33.69) |  |
| West | 809(25.55) | 116(24.42) |  |
| **Elixhauser comorbidity score** | 3.06(1.84) | 3.38(1.80) | 0.0004 |
| **Primary diagnosis** |  |  | <0.0001 |
| Cancer-related disorders | 1775(56.06) | 172(36.21) |  |
| Infections | 453(14.31) | 119(25.05) |  |
| Genitourinary disorders | 60(1.90) | 12(2.53) |  |
| Cardiovascular disorders | 172(5.43) | 40(8.42) |  |
| Pulmonary disorders | 355(11.21) | 61(12.84) |  |
| Gastrointestinal disorders | 179(5.65) | 31(6.53) |  |
| Fractures | * | * |  |
| Fluid/Electrolyte disorders | 11(0.35) | * |  |
| Neurologic disorders | 31(0.98) | 3(0.63) |  |
| Complications of surgery | 64(2.02) | 15(3.16) |  |
| Other disorders | 61(1.93) | 13(2.74) |  |
| **Cancer type** |  |  | <0.0001 |
| Ovarian cancer | 2079(65.67) | 241(50.74) |  |
| Uterine cancer | 725(22.90) | 162(34.11) |  |
| Cervical cancer | 362(11.43) | 72(15.15) |  |
| **Metastatic sites** |  |  |  |
| Bone & bone marrow | 205(6.48) | 51(10.74) | 0.0007 |
| Brain & spinal cord | 153(4.83) | 31(6.53) | 0.1161 |
| Lymph nodes | 502(15.86) | 62(13.05) | 0.1153 |
| Liver | 602(19.01) | 117(24.63) | 0.0041 |
| Respiratory organs | 972(30.70) | 166(34.95) | 0.0626 |
| Adrenal glands | 24(0.76) | * | 0.5006 |
| Gastrointestinal organs | 1808(57.11) | 241(50.74) | 0.0091 |
| Urinary organs | 187(5.91) | 22(4.63) | 0.2653 |
| Genital organs | 377(11.91) | 24(5.05) | <0.0001 |
| Other organs | 608(19.20) | 77(16.21) | 0.1195 |
| **Number of metastatic sites (≥2)** | 1441(45.51) | 217(45.68) | 0.9449 |
| **Type of CCT** |  |  |  |
| PEG tube | 71(2.24) | 13(2.74) | 0.5034 |
| TPN | 476(15.03) | 60(12.63) | 0.1681 |
| Tracheostomy | 185(5.84) | 23(4.84) | 0.3806 |
| AKI requiring dialysis | 154(4.86) | 28(5.89) | 0.3365 |
| **Do Not Resuscitate** | 186(5.87) | 201(42.32) | <0.0001 |
| **Chemotherapy** | 220(6.95) | 30(6.32) | 0.6109 |
| **In-hospital mortality** | 1325(41.85) | 333(70.11) | <0.0001 |
| **Discharge disposition (alive)** |  |  |  |
| Home or home healthcare | 992(53.89) | 45(31.69) | <0.0001 |
| Short term hospitals | 96(5.21) | * |  |
| Intermediate facilities | 744(40.41) | 86(60.56) |  |
| Other | * | * |  |

Abbreviation: CCT, critical care therapies; mGCa, metastatic gynecologic cancer; PC, palliative care; SD, standard deviation; IMV, invasive mechanic ventilation; PEG, percutaneous endoscopic gastrostomy; TPN, total parenteral nutrition; AKF, acute kidney failure;

* Small numbers of observations (<10) are at risk of identification of persons according to the HUCP and we replaced the number with an asterisk.


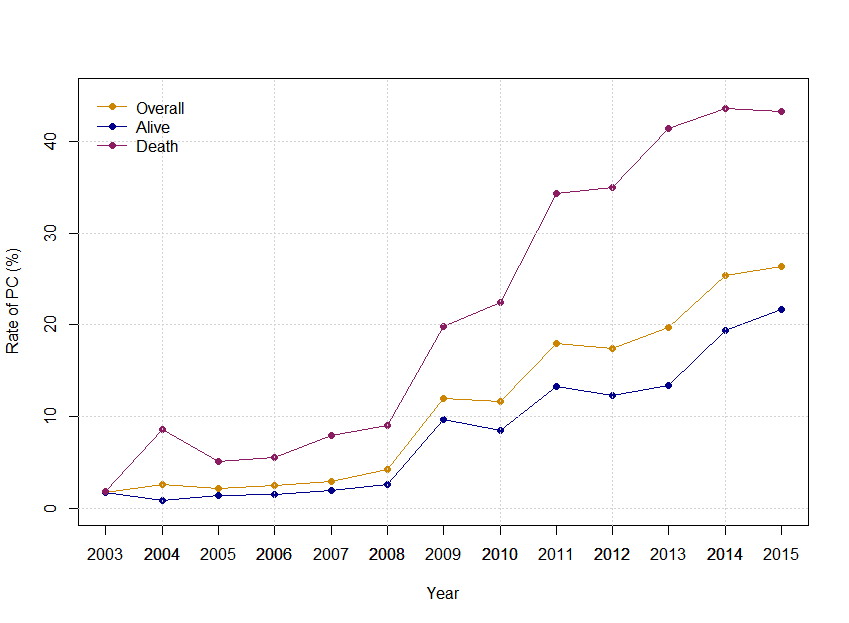


Supplementary Figure 1. Inpatient palliative care referral over time, stratified by discharge destination.
